# Right ventricular free-wall strain-based risk stratification for temporary mechanical circulatory support in cardiogenic shock

**DOI:** 10.1093/eschf/xvag116

**Published:** 2026-04-27

**Authors:** Chong Bin Lee, Andreas Merz, Daniel-Armando Morris, Matthias Schneider-Reigbert, Linus Haberbosch, Fabian Spinka, Caroline Büttner, Cheng-Ying Chiu, Ingo Hilgendorf, Robin Kraft, Thomas Schlabs, Athanasios Frydas

**Affiliations:** Deutsches Herzzentrum der Charité, Department of Cardiology, Angiology and Intensive Care Medicine, Campus Virchow-Klinikum, Mittelallee 11, Berlin 13353, Germany; Charité—Universitätsmedizin Berlin, Corporate Member of Freie Universität Berlin and Humboldt-Universität zu Berlin, Augustenburger Platz 1, 13353 Berlin, Germany; Structural Heart Interventions Program (SHIP), Deutsches Herzzentrum der Charité, Augustenburger Platz 1, Berlin 13353, Germany; DZHK (German Center for Cardiovascular Research) Partner Site Berlin, Berlin, Germany; Deutsches Herzzentrum der Charité, Department of Cardiology, Angiology and Intensive Care Medicine, Campus Virchow-Klinikum, Mittelallee 11, Berlin 13353, Germany; Charité—Universitätsmedizin Berlin, Corporate Member of Freie Universität Berlin and Humboldt-Universität zu Berlin, Augustenburger Platz 1, 13353 Berlin, Germany; DZHK (German Center for Cardiovascular Research) Partner Site Berlin, Berlin, Germany; Deutsches Herzzentrum der Charité, Department of Cardiology, Angiology and Intensive Care Medicine, Campus Virchow-Klinikum, Mittelallee 11, Berlin 13353, Germany; Charité—Universitätsmedizin Berlin, Corporate Member of Freie Universität Berlin and Humboldt-Universität zu Berlin, Augustenburger Platz 1, 13353 Berlin, Germany; DZHK (German Center for Cardiovascular Research) Partner Site Berlin, Berlin, Germany; Deutsches Herzzentrum der Charité, Department of Cardiology, Angiology and Intensive Care Medicine, Campus Virchow-Klinikum, Mittelallee 11, Berlin 13353, Germany; Charité—Universitätsmedizin Berlin, Corporate Member of Freie Universität Berlin and Humboldt-Universität zu Berlin, Augustenburger Platz 1, 13353 Berlin, Germany; DZHK (German Center for Cardiovascular Research) Partner Site Berlin, Berlin, Germany; Charité—Universitätsmedizin Berlin, Corporate Member of Freie Universität Berlin and Humboldt-Universität zu Berlin, Augustenburger Platz 1, 13353 Berlin, Germany; Deutsches Herzzentrum der Charité, Department of Cardiology, Angiology and Intensive Care Medicine, Campus Virchow-Klinikum, Mittelallee 11, Berlin 13353, Germany; Charité—Universitätsmedizin Berlin, Corporate Member of Freie Universität Berlin and Humboldt-Universität zu Berlin, Augustenburger Platz 1, 13353 Berlin, Germany; DZHK (German Center for Cardiovascular Research) Partner Site Berlin, Berlin, Germany; Deutsches Herzzentrum der Charité, Department of Cardiology, Angiology and Intensive Care Medicine, Campus Virchow-Klinikum, Mittelallee 11, Berlin 13353, Germany; Charité—Universitätsmedizin Berlin, Corporate Member of Freie Universität Berlin and Humboldt-Universität zu Berlin, Augustenburger Platz 1, 13353 Berlin, Germany; Deutsches Herzzentrum der Charité, Department of Cardiology, Angiology and Intensive Care Medicine, Campus Virchow-Klinikum, Mittelallee 11, Berlin 13353, Germany; Charité—Universitätsmedizin Berlin, Corporate Member of Freie Universität Berlin and Humboldt-Universität zu Berlin, Augustenburger Platz 1, 13353 Berlin, Germany; DZHK (German Center for Cardiovascular Research) Partner Site Berlin, Berlin, Germany; Deutsches Herzzentrum der Charité, Department of Cardiology, Angiology and Intensive Care Medicine, Campus Virchow-Klinikum, Mittelallee 11, Berlin 13353, Germany; Charité—Universitätsmedizin Berlin, Corporate Member of Freie Universität Berlin and Humboldt-Universität zu Berlin, Augustenburger Platz 1, 13353 Berlin, Germany; DZHK (German Center for Cardiovascular Research) Partner Site Berlin, Berlin, Germany; Deutsches Herzzentrum der Charité, Department of Cardiology, Angiology and Intensive Care Medicine, Campus Virchow-Klinikum, Mittelallee 11, Berlin 13353, Germany; Charité—Universitätsmedizin Berlin, Corporate Member of Freie Universität Berlin and Humboldt-Universität zu Berlin, Augustenburger Platz 1, 13353 Berlin, Germany; DZHK (German Center for Cardiovascular Research) Partner Site Berlin, Berlin, Germany; Deutsches Herzzentrum der Charité, Department of Cardiology, Angiology and Intensive Care Medicine, Campus Virchow-Klinikum, Mittelallee 11, Berlin 13353, Germany; Charité—Universitätsmedizin Berlin, Corporate Member of Freie Universität Berlin and Humboldt-Universität zu Berlin, Augustenburger Platz 1, 13353 Berlin, Germany; DZHK (German Center for Cardiovascular Research) Partner Site Berlin, Berlin, Germany; Deutsches Herzzentrum der Charité, Department of Cardiology, Angiology and Intensive Care Medicine, Campus Virchow-Klinikum, Mittelallee 11, Berlin 13353, Germany; Charité—Universitätsmedizin Berlin, Corporate Member of Freie Universität Berlin and Humboldt-Universität zu Berlin, Augustenburger Platz 1, 13353 Berlin, Germany; Department of Cardiology, University Hospital Zurich, Raemistrasse 100, 8091 Zurich, Switzerland

**Keywords:** Right ventricular strain, Cardiogenic shock, Intensive care medicine, Mechanical circulatory support

## Abstract

**Introduction:**

Early identification of patients with cardiogenic shock (CS) who will require temporary mechanical circulatory support (MCS) remains challenging. Right ventricular (RV) dysfunction is common in CS and affects haemodynamic stability. RV free wall longitudinal strain (RV FWLS) is a sensitive marker of myocardial dysfunction, but its role in predicting MCS escalation in CS remains unclear.

**Methods:**

In this single-centre retrospective study, patients admitted with CS between January 2023 and December 2025 were screened. Inclusion required transthoracic echocardiography within 24 h of CS diagnosis and prior to MCS implantation. RV FWLS was measured using commercially available software. Primary outcome was temporary MCS implantation during hospitalization. Secondary outcomes included in-hospital mortality and intensive care and hospital length of stay.

**Results:**

Ninety-two patients were included; 31 (34%) required temporary MCS. Severe RV FWLS impairment (<11%) was strongly associated with temporary MCS use (OR 10.49, 95% CI 3.72–29.59). Tricuspid annular plane systolic excursion and fractional area change were not significantly associated with temporary MCS. Severe RV FWLS was linked to longer intensive care stay (21 vs 8 days, *P* = .003) and hospital stay (25 vs 14 days, *P* = .003), but not mortality. RV FWLS demonstrated moderate discrimination (area under the curve, AUC 0.74), improving with left ventricular ejection fraction (LVEF) and Sequential Organ Failure Assessment (SOFA) score (AUC 0.82). A classification and regression tree -derived algorithm using RV FWLS, SOFA score, and LVEF stratified patients into distinct risk groups with 78% overall accuracy and 95% specificity.

**Conclusion:**

Integration of RV FWLS with clinical parameters may improve early risk stratification in CS.

## Graphical Abstract

**Graphical Abstract:**
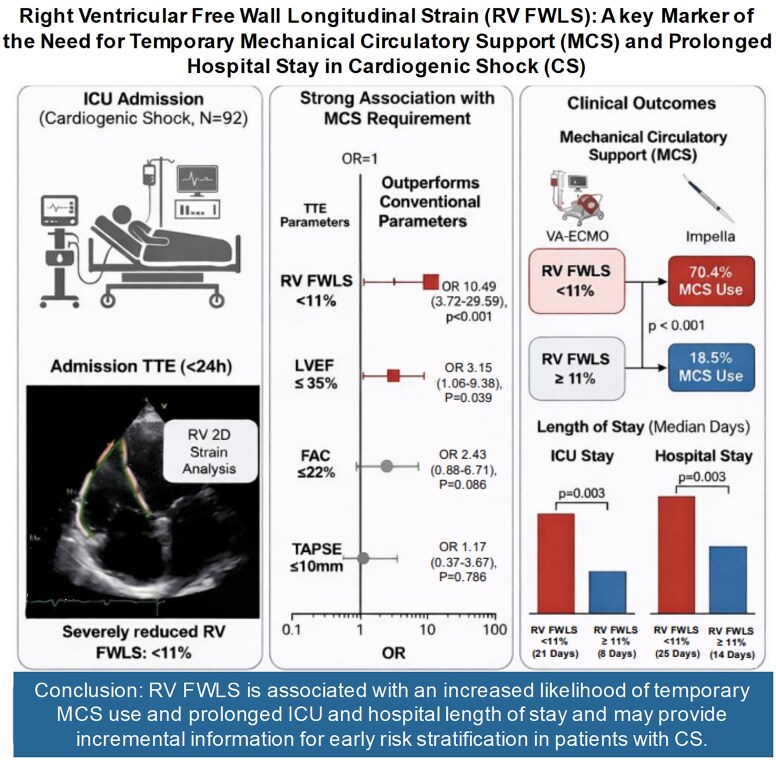

## Introduction

Temporary mechanical circulatory support (MCS) is increasingly utilized cardiogenic shock (CS), with expanding indications and demonstrated survival benefits, particularly in high-volume experienced centres. While some patients need immediate support, others initially stabilize and deteriorate later. Early risk stratification before overt haemodynamic collapse therefore represents an important unmet clinical need.^1–5^

Right ventricular (RV) dysfunction is a major determinant of prognosis in CS. Biventricular failure is common in CS, with RV dysfunction observed in 44% of patients with acute myocardial infarction-related CS, and even higher rates in non-ischaemic CS.^2,3^ RV free wall longitudinal strain (RV FWLS) has emerged as a sensitive marker of myocardial dysfunction and often outperforms conventional measures such as tricuspid annular plane systolic excursion (TAPSE) and fractional area change (FAC) in heart failure populations.^6^

The potential advantages of strain imaging over TAPSE are particularly relevant in the intensive care setting. TAPSE is influenced by loading conditions, interventricular dependence, and prior cardiac surgery, which may lead to misleading estimates of RV function.^7^ In contrast, strain-derived parameters quantify active myocardial deformation and are less affected by passive motion and translational artefacts.^8,9^ With increasing automation and user-friendly software, strain analysis has become more feasible for routine clinical use.^9^

RV strain has been identified as predictive of outcomes in acute myocardial infarction and has been shown to be a predictor of successful microaxial flow pump weaning. Despite the robust prognostic value of RV strain in heart failure populations, data specifically examining RV strain in CS patients remains very limited.^6,10–15^ Its role in guiding decisions regarding MCS escalation in the broader CS population has not been well defined.^6,16,17^

### Objective

The objective of this study was to evaluate the association between RV strain parameters and (i) the need for temporary MCS and (ii) clinical outcomes in patients with CS.

## Methods

### Study design and population

We conducted a retrospective, single-centre observational study of all consecutive patients admitted to the intensive care unit (ICU) of our tertiary-care, high-volume cardiac centre with a diagnosis of CS between January 2023 and December 2025. A total of 355 consecutive patients were screened for eligibility through a search of our institutional database using the ICD code for CS.

CS was defined according to the Society for Cardiovascular Angiography and Interventions (SCAI) classification system (stages A-E). Patients were classified based on haemodynamic status, markers of hypoperfusion, and use of advanced circulatory support.^2,18^ SCAI stage B patients had either isolated hypoperfusion (lactate 2–5 mmol/L) or hypotension (systolic blood pressure 60–90 mm Hg or mean arterial pressure 50–65 mm Hg) without the need for pharmacological or device therapy. SCAI stage C patients had hypoperfusion and hypotension or were being treated with one vasopressor/inotrope or one circulatory support device. SCAI stage D patients had persistent hypotension and hypoperfusion (lactate 5–10 mmol/L) despite treatment or required 2–5 drugs or devices. SCAI stage E patients had severe hypotension (systolic blood pressure 60 mm Hg or mean arterial pressure 50 mm Hg) or severe hypoperfusion (lactate ≥10 mmol/L or pH ≤7.2) or required ≥3 drugs or ≥3 devices.^18^

CS aetiologies were classified according to a previously published classification system for clinical research.^19^ CS was categorized as: (i) Acute myocardial infarction-CS, defined as CS in the setting of acute myocardial infarction (ii) HF-CS, defined as primary left or RV failure in the absence of acute myocardial infarction, and (iii) secondary CS, defined as CS due to a primary non-myocardial cardiac cause, such as arrhythmia, pericardial disease, or valvular disease.

The indication and selection of temporary MCS were determined according to the established clinical routine of our centre. This approach is standardized and is regulated by a formal standard operating procedure, which has been previously published.^20^ MCS was considered in cases of ongoing chest compressions and pharmacological resuscitation, as well as in patients with severely reduced LVEF accompanied by rapidly deteriorating haemodynamic status, progressive worsening of organ function, and metabolic derangement despite escalating doses of inotropic and vasoactive support (vasoactive-inotropic score [VIS] > 20).

### Inclusion and exclusion criteria

Patients were included if a transthoracic echocardiogram (TTE) was performed within the first 24 h of the CS diagnosis.

Patients were excluded for the following reasons:

- TTE performed later than 24 h after ICU admission
- TTE obtained only after the implantation of MCS
- heart rate >120 beats per minute at the time of image acquisition (tachycardia substantially limits the methodological validity of speckle tracking analysis)
- insufficient acoustic windows or inadequate visualization of the RV free wall preventing strain analysis
- MCS implantation for ‘protected’ interventions
- patients with CS after cardiac surgery

### Echocardiographic acquisition

All TTEs were performed according to current American Society of Echocardiography (ASE) and European Association of Cardiovascular Imaging guidelines using commercially available ultrasound systems.^8^ Standard apical, parasternal, and subcostal views were obtained. RV strain analysis was performed using an RV-focused apical four-chamber view, ensuring that the entire RV free wall was clearly visualized and foreshortening was minimized.^8,21^

### Image analysis and strain measurements

All examinations were analysed offline using TomTec Arena (TomTec Imaging Systems, Munich, Germany). One senior echocardiographer performed all RV measurements while blinded to all clinical and laboratory data.

For patients in sinus rhythm, one representative cardiac cycle with optimal endocardial tracking quality was selected for analysis. For patients in atrial fibrillation, three consecutive cardiac cycles were analysed, and the mean values were reported.^8^

RV strain analysis was performed retrospectively and was not available to the treating clinicians during patient management.

### Assessment of RV dysfunction

RV systolic dysfunction was assessed using conventional and deformation-based echocardiographic parameters in accordance with current ASE recommendations.^8^ Severe RV dysfunction was defined using guideline-based cutoffs as follows: RV longitudinal free wall strain <11%, RV global longitudinal strain ≤9%, FAC ≤ 22%, and TAPSE ≤ 10 mm. These thresholds correspond to the severe abnormality range for RV systolic function as proposed by the ASE expert consensus document.^8^ Left ventricular systolic dysfunction was classified as severe according to standard guideline-based left ventricular ejection fraction (LVEF ≤35%) thresholds. Absolute strain values were used for all deformation parameters.

### Clinical and laboratory data

Clinical information, including demographics, comorbidities, haemodynamic parameters, SCAI shock stage, and medication use (including doses and numbers of vasopressors/inotropes), was extracted from electronic medical records. Laboratory data at baseline included markers of end-organ perfusion (lactate, creatinine, bilirubin), inflammatory markers, and cardiac biomarkers (high-sensitivity troponin, N-terminal pro-brain natriuretic peptide).

### Follow-up and outcomes

The primary outcome was the need for temporary MCS implantation during the index hospitalization due to clinical or haemodynamic deterioration. Secondary outcomes included in-hospital mortality, duration of ICU- and in-hospital stay. MCS devices included veno-arterial extracorporeal membrane oxygenation, Impella CP, Impella 5.5, intra-aortic balloon pump, temporary right ventricular assist device, and combined support strategies. Durable left ventricular assist device (LVAD) implantation was not considered a form of temporary MCS in our analysis. Duration of hospitalization was defined as the total length of the index admission. Patients requiring durable LVAD implantation due to lack of recovery were transferred to the cardiac surgery department and their ICU and hospital stay included the complete treatment period.

## Results

**Figure 1:**
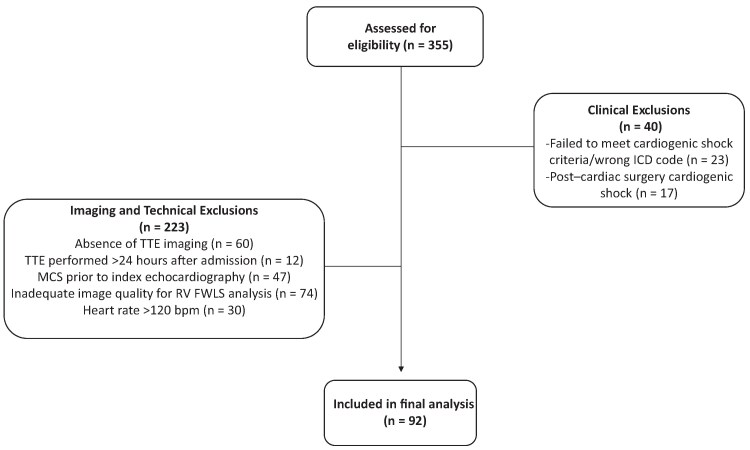
Study flow diagram. Flow chart of patient screening and selection. Of 355 screened patients, 92 were included in the final analysis after predefined clinical and imaging/technical exclusions. Abbreviations: CS, cardiogenic shock; ICD, International Classification of Diseases; MCS, mechanical circulatory support; RV FWLS, right ventricular free wall longitudinal strain; TTE, transthoracic echocardiography

A total of 355 patients were screened for eligibility. Overall, 263 patients were excluded, including 39 for clinical reasons and 224 for imaging or technical reasons (*Figure 1*). A comparison of baseline characteristics between included and excluded patients is provided in Supplementary Material S1. The baseline and echocardiographic characteristics of the study cohort are shown in *Table 1* and *Table 2*. Baseline characteristics in patients with and without severely reduced RV FWLS are presented in Supplementary Material S2.

**Table 1:** Baseline characteristics

Parameters	Patients with cardiogenic shock (*n* = 92)
**Baseline clinical status**	
Age, years	65 (52–76)
Male	70 (76)
SOFA Score	7 (4–11)
GCS	15 (13–15)
Mechanical ventilation	14 (15)
Dialysis	4 (4)
Septic shock	14 (15)
Haemorrhagic shock	1 (1)
Pulmonary embolism	5 (5)
**Past medical history**	
Arterial hypertension	48 (52)
Diabetes	27 (29)
Dyslipidemia	45 (49)
Coronary artery disease	45 (49)
Myocardial infarction	33 (36)
CABG	6 (7)
Other cardiac surgery	9 (10)
Pacemaker	25 (27)
Atrial fibrillation	23 (25)
COPD	14 (15)
**Drug therapy**	
ARNI	27 (29)
ACE-I/ARB	26 (28)
Betablocker	52 (57)
Diuretics	41 (45)
SGLT2-I	33 (36)
MRA	31 (34)
**Laboratory**	
Creatinine, mg/dl	1.7 (1.1–2.4)
Lactate at admission, mg/dl	24 (14–46)
Lactate during echocardiography, mg/dl	22 (14–41)
Troponine, ng/l	91 (48–253) (*n* = 69)
NTproBNP, ng/l	13 317 (4014–30 034) (*n* = 69)
Bilirubin, mg/dl	1.1 (0.9–1.7)
**Mechanism of cardiogenic shock**	
LV failure	79 (86)
RV failure	41 (45)
Biventricular failure	33 (36)
Acute myocardial infarction cardiogenic shock	22 (24)
Heart failure cardiogenic shock	54 (59)
Secondary cardiogenic shock	16 (17)
**Classification of cardiogenic shock**	
SCAI A	2 (2)
SCAI B	15 (16)
SCAI C	43 (47)
SCAI D	22 (24)
SCAI E	10 (11)

Data are presented in median (Q1-Q3) and in *n* (%).

ACE-I, Angiotensin-Converting Enzyme Inhibitor; ARB, Angiotensin II Receptor Blocker; ARNI, Angiotensinrezeptor-Neprilysin-Inhibitor; CABG, coronary artery bypass graft; COPD, chronic obstructive pulmonary disease; GCS, Glasgow Coma Sale; MRA, Mineralocorticoid Receptor Antagonist; NTproBNP, N-Terminal pro-B-type Natriuretic Peptide; LV, left ventricle; RV, right ventricle; SCAI, Society for Cardiovascular Angiography and Interventions; SGLT2-I, Sodium-Glucose Cotransporter 2 Inhibitor; SOFA, sequential organ failure assessment score.

**Table 2:** Echocardiographic parameters

Parameters	Patients with cardiogenic shock (*n* = 92)
**Left ventricle**	
LVEF, %	28 (19–39) (*n* = 88)
LV average GLS, %	6.2 (4.3–9.9) (*n* = 69)
LVEDD, mm	55 (48–62) (*n* = 74)
LVEDV, ml	170 (107–220) (*n* = 80)
**Left atrium**	
Left atrial volume, ml	82 (66–118) (*n* = 73)
Left atrial average GLS, %	8.5 (5.8–13.3) (*n* = 61)
**Right ventricle**	
RV diameter, mm	43 (37–49)
RV GLS, %	11.5 (7.2–16.1)
RV free wall strain, %	15.1 (10.6–19.7)
TAPSE, mm	15 (11–18) (*n* = 78)
RV FAC, %	32 (23–39) (*n* = 89)
**Valvular disease**	
Severe aortic stenosis	3/73 (4)
Severe aortic regurgitation	1/80 (1)
Severe mitral regurgitation	6/76 (8)
Severe tricuspid regurgitation	14/70 (20)

Data are presented in median (Q1-Q3) and in *n* (%).

FAC, fractional area change; GLS, global longitudinal strain; LVEDD, left ventricular end-diastolic diameter; LVEF, left ventricular ejection fraction; LVEDV, left ventricular end-diastolic volume; RV, right ventricle; TAPSE, tricuspid annular plane systolic excursion.

Overall, the cohort represented a severely ill population with advanced CS. Median SOFA score was 7 (4–11), median lactate at admission was 24 mg/dl (14–46), and median creatinine was 1.7 mg/dl (1.1–2.4). Temporary MCS was required in 31 patients (34%). Median ICU length of stay was 11 days (5–22). In-hospital mortality occurred in 31 patients (34%), with CS being the leading cause of death. Detailed clinical outcomes are reported in *Table 3*.

**Table 3:** Clinical outcomes

Parameters	Patients with cardiogenic shock (*n* = 92)
**Mechanical circulatory support**	
MCS use	31 (34)
Successful MCS weaning	17/31 (55)
Impella 5.5	20 (22)
Successful Impella 5.5 weaning	11/20 (55)
Impella CP	9 (10)
Successful Impella CP weaning	6/9 (67)
VA-ECMO	13 (14)
Successful VA-ECMO weaning	10/13 (77)
Temporary RVAD	4 (4)
Successful RVAD weaning	1/4 (25)
IABP	1 (1)
Successful IABP weaning	1/1 (100)
Combined Impella and VA-ECMO	11 (12)
**Intra-hospital outcomes**	
Dialysis	36 (39)
Pacemaker implantation	8 (9)
Septic shock	20 (22)
Mechanical ventilation	41 (45)
Stroke	6 (7)
Intracranial bleeding	3 (3)
ICU stay, days	11 (5–22)
In-hospital stay, days	17 (9–30)
Discharge home from index hospital	31 (34)
Durable LVAD	12 (13)
Intra-hospital death	31 (34)
Death due to deterioration of cardiogenic shock	25 (27)
Death due to bleeding	1 (1)
Death due to sepsis	7 (8)
Death due to brain hypoxia	4 (4)
**Short- and midterm outcome**	
30-day mortality	27/91 (30)
Re-admission due to heart failure after 3months	**9/38 (24)**
Re-admission due to heart failure after 6months	**7/31 (23)**

Data are presented in median (Q1–Q3) and in *n* (%).

IABP, intra-aortic balloon pump; LVAD, left ventricular assist device; MCS, mechanical circulatory support; RVAD, right ventricular assist device; VA-ECMO, veno-arterial extracorporeal membrane oxygenation.

### Association of RV function with temporary MCS use

In univariable logistic regression analyses, RV strain–based parameters were strongly associated with temporary MCS use. Severe impairment of RV FWLS was associated with a markedly increased likelihood of temporary MCS use (odds ratio [OR] 10.49, 95% confidence interval [CI] 3.72–29.59). Similarly, severe impairment of RV GLS was associated with a significantly higher odds of temporary MCS use (OR 6.71, 95% CI 2.57–17.51).

In contrast, conventional RV functional parameters were not significantly associated with temporary MCS use. FAC ≤22% showed no significant association (OR 2.43, 95% CI 0.88–6.71), and TAPSE ≤10 mm was likewise not associated with temporary MCS use (OR 1.17, 95% CI 0.37–3.67). Severely reduced LVEF was associated with a three-fold increased odds of temporary MCS use (OR 3.15, 95% CI 1.06–9.38).

**Figure 2:**
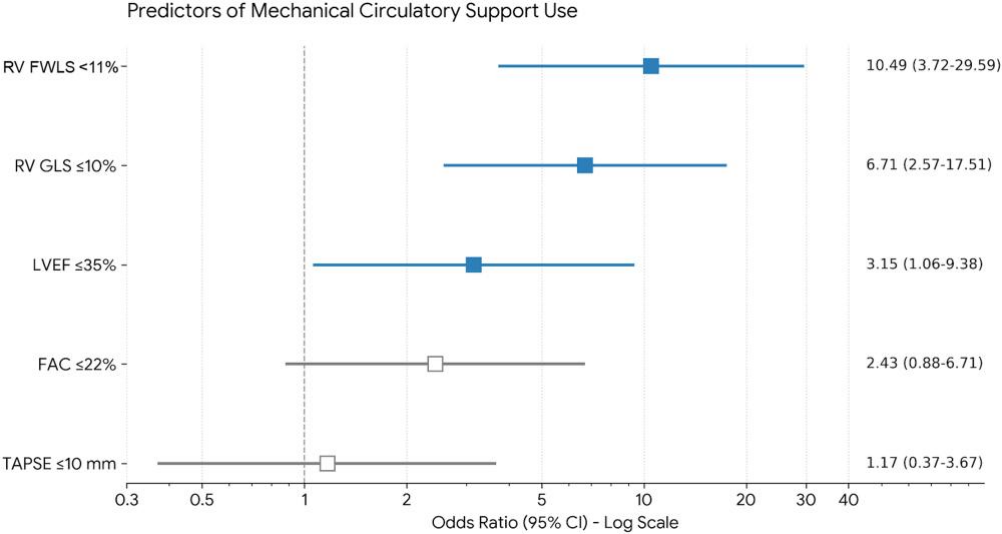
Odds ratios for mechanical circulatory support use according to ventricular functional parameters. Forest plot showing odds ratios (ORs) and 95% confidence intervals (CIs) for the association between echocardiographic ventricular functional parameters and temporary mechanical circulatory support (MCS) use derived from univariable logistic regression analyses. Severe right ventricular (RV) free wall longitudinal strain impairment and severe RV global longitudinal strain impairment were strongly associated with temporary MCS use, whereas conventional RV functional parameters, including fractional area change (FAC ≤22%) and tricuspid annular plane systolic excursion (TAPSE ≤10 mm), were not. Severe left ventricular systolic dysfunction was also associated with increased odds of temporary MCS use. RV strain and FAC analyses were based on 92 patients, whereas TAPSE measurements were available in 79 patients

Odds ratios and confidence intervals for predictors of temporary MCS use are shown in *Figure 2*.

In multivariable logistic regression analysis including RV FWLS, SOFA score, and LVEF (model size guided by the number of MCS events), RV FWLS remained independently associated with temporary MCS use (OR 0.865 per 1% increase, *P* = .005). The SOFA score was also independently associated with temporary MCS use (OR 1.214 per point increase, *P* = .010), whereas LVEF was not independently associated (*P* = .129). In an additional multivariable logistic regression analysis including CS aetiology, severe RV FWLS remained independently associated with the need for temporary MCS (OR 15.27, *P* < .001), whereas shock aetiology was not a significant predictor (OR 0.55, *P* = .183).

### Association of RV free wall strain with echocardiographic and clinical parameters

RV FWLS showed a very strong correlation with RV GLS (ρ = 0.93, *P* < .001) and strong correlations with conventional RV systolic function parameters, including FAC (ρ = 0.73, *P* < .001) and TAPSE (ρ = 0.62, *P* < .001). RV FWLS was inversely correlated with RV diameter (ρ = −0.35, *P* < .001).

Moderate correlations were observed between RV free wall strain and left ventricular mechanics, including LV GLS (ρ = 0.55, *P* < .001) and LVEF (ρ = 0.40, *P* < .001), reflecting ventricular interdependence. In contrast, RV FWLS was not significantly correlated with lactate, or creatinine.

### Discriminative performance of RV strain for temporary MCS use

Receiver operating characteristic (ROC) curve analysis demonstrated moderate discriminative ability of RV FWLS for identifying patients requiring temporary MCS, with an area under the curve (AUC) of 0.74 (95% CI 0.63–0.85, *P* < .001). In contrast, both baseline LVEF and Sequential Organ Failure Assessment (SOFA) score showed weaker discriminative performance, with AUCs of 0.62 (95% CI 0.50–0.74, *P* = .054) and 0.65 (95% CI 0.53–0.78, *P* = .013), respectively.

**Figure 3:**
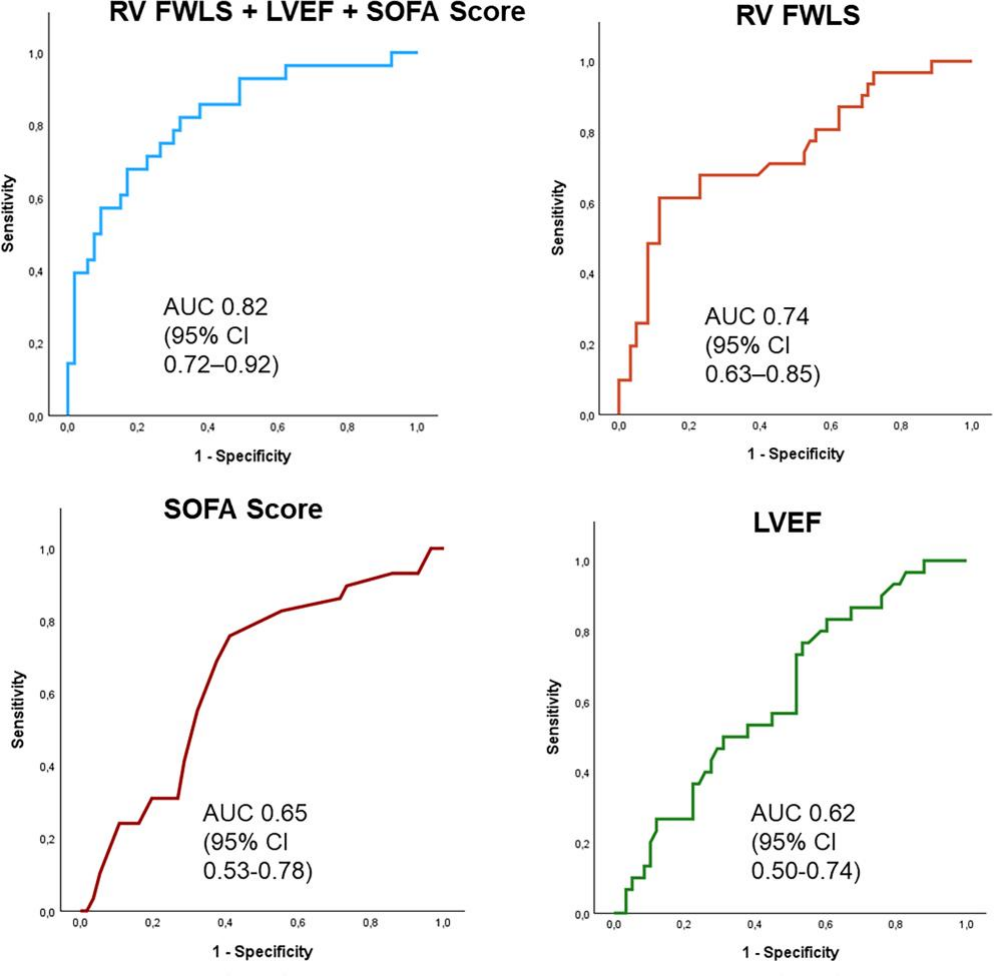
Receiver operating characteristic (ROC) curves demonstrating the discriminative performance of right ventricular free wall longitudinal strain (RV FWLS), left ventricular ejection fraction (LVEF), SOFA score, and a combined predictive model for temporary mechanical circulatory support (MCS) use. RV FWLS showed moderate discrimination for temporary MCS requirement (AUC 0.74, 95% CI 0.63–0.85), whereas LVEF (AUC 0.62, 95% CI 0.50–0.74) and SOFA score (AUC 0.65, 95% CI 0.53–0.78) demonstrated weaker discriminative performance. A combined model incorporating RV FWLS, LVEF, and SOFA score yielded improved discrimination (AUC 0.82, 95% CI 0.72–0.92)

A combined model incorporating RV FWLS, LVEF, and SOFA score yielded improved discriminative performance, with an AUC of 0.82 (95% CI 0.72–0.92, *P* < .001), indicating superior risk stratification compared with any single parameter alone. ROC curves for individual predictors and the combined model are shown in *Figure 3*.

### Algorithmic risk stratification using classification and regression tree analysis

To enhance clinical applicability, a classification and regression tree (CART) analysis was performed using predefined dichotomized predictors: RV FWLS <11%, SOFA score (≥7), and LVEF ≤35%. RV free wall strain emerged as the primary discriminator of temporary MCS use. Patients with RV FWLS <11% had a substantially higher likelihood of requiring temporary MCS compared with those with preserved RV strain. Subsequent stratification by SOFA score further refined risk, identifying a highest-risk subgroup characterized by both impaired RV strain and elevated SOFA scores. LVEF ≤35% provided additional, more limited refinement of risk within selected subgroups.

**Figure 4:**
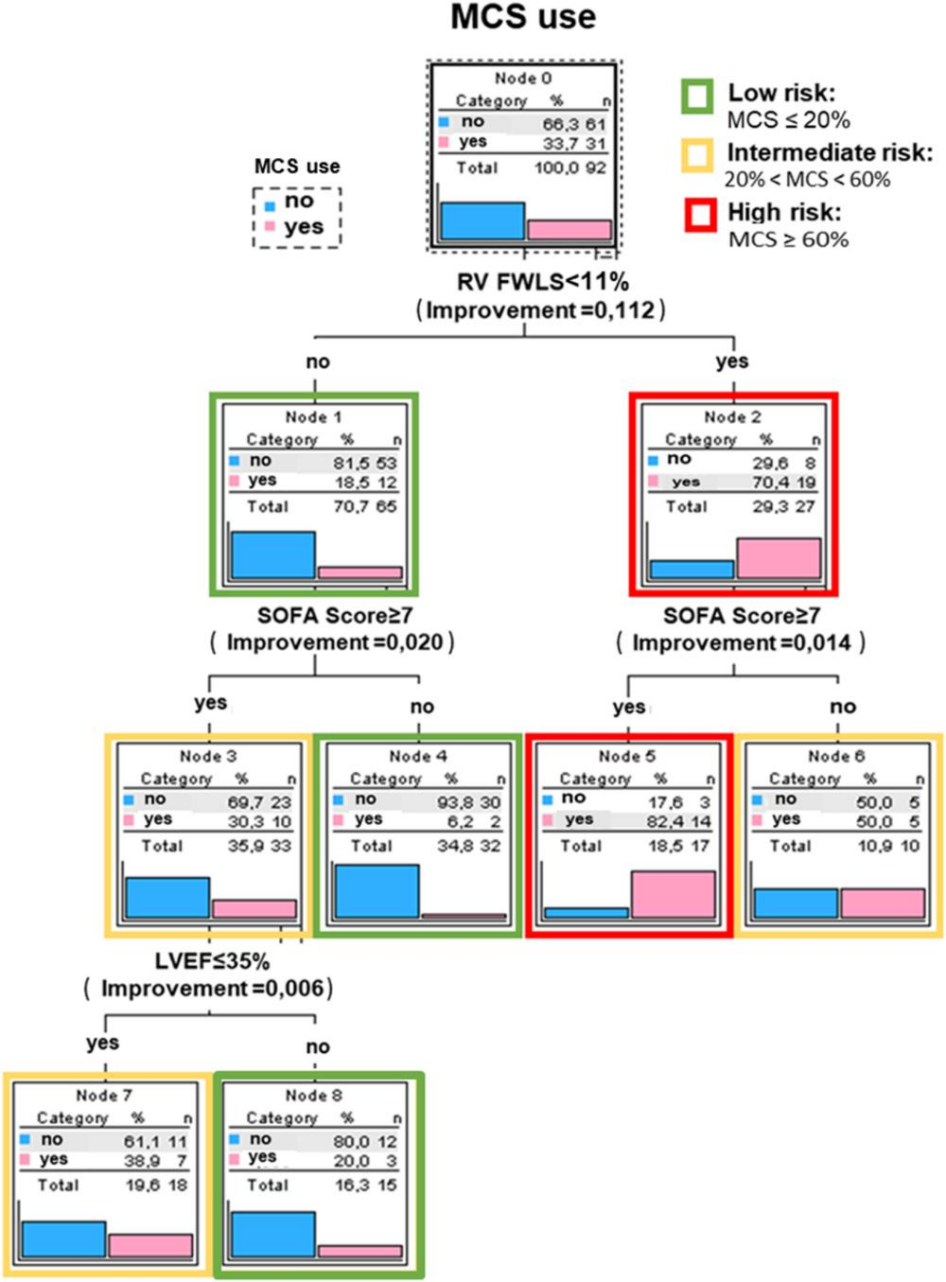
Classification and regression tree (CART)-derived decision algorithm for prediction of temporary mechanical circulatory support (MCS) use. The model was constructed using predefined dichotomized predictors: right ventricular free wall longitudinal strain (RV FWLS <11%), SOFA score (≥7), and left ventricular ejection fraction (LVEF ≤35%). RV FWLS emerged as the primary discriminator of temporary MCS risk, with subsequent stratification by clinical severity and left ventricular systolic function. Terminal nodes display the proportion of patients requiring temporary MCS within each risk group. The tree was validated using cross-validation (resubstitution risk 0.217; cross-validated risk 0.261 ± 0.046)

The CART-derived algorithm stratified patients into clinically distinct risk categories, with observed MCS rates ranging from approximately 6% in the lowest-risk group to over 80% in the highest-risk group. Overall classification accuracy was 78%, with high specificity (95%) and moderate sensitivity (45%), and performance was maintained with cross-validation. The final CART model demonstrated a resubstitution risk of 0.217 and a cross-validated risk of 0.261 (SE 0.046), corresponding to an expected misclassification rate of approximately **26%** in unseen data. The decision tree is shown in *Figure 4*.

### Clinical outcomes according to RV function

Patients with severe RV FWLS were substantially more likely to require temporary MCS compared with those without severe RV FWLS impairment (70.4% vs 18.5%, *P* < .001). Severe RV FWLS impairment was also associated with significantly prolonged ICU and hospital length of stay. Median ICU stay was 21 days (interquartile range [IQR] 11.5–32.5) in patients with severe RV FWLS impairment versus 8 days (IQR 4–18) in those without (*P* = .003), while median hospital length of stay was 25 days (IQR 15.5–38.5) versus 14 days (IQR 8–25), respectively (*P* = .003).

When hospital length of stay was analysed across RV functional parameters, strain-based measures remained significantly associated with prolonged hospitalization. Patients with severe RV FWLS impairment had longer hospital stays than those without severe involvement (mean rank 59.5 vs 41.1; Mann–Whitney U = 526, *P* = .003), with a similar association observed for severe RV global longitudinal strain (mean rank 60.6 vs 38.6; Mann–Whitney U = 507.5, *P* < .001). In contrast, hospital length of stay did not differ significantly according to conventional RV parameters, including TAPSE ≤10 mm (*P* = .795) and FAC ≤22% (*P* = .053), nor according to severely reduced LVEF (*P* = .128).

In-hospital mortality did not differ significantly between patients with and without severe RV FWLS impairment (25.9% vs 36.9%, *P* = .31). Clinical outcomes for the overall cohort and stratified by RV FWLS severity are summarized in *Table 3* and *Table 4* and Supplementary Material S3.

**Table 4:** Association of right ventricular free wall strain with clinical outcomes

Outcome	RV free wall strain ≥11% (*n* = 65)	RV free wall strain <11% (*n* = 27)	*P* value
MCS use, *n* (%)	12 (18.5%)	19 (70.4%)	<.001
ICU length of stay, days	8 (4–18)	21 (11.5–32.5)	.003
Hospital length of stay, days	14 (8–25)	25 (15.5–38.5)	.003
In-hospital mortality, *n* (%)	24 (36.9%)	7 (25.9%)	.31

Values are presented as *n* (%) or median (interquartile range). Comparisons between groups were performed using the Mann–Whitney U test for continuous variables and χ^2^ or Fisher’s exact test for categorical variables, as appropriate.

### Mortality analyses

In univariable logistic regression analyses, none of the assessed RV functional parameters nor severe left ventricular systolic dysfunction were significantly associated with in-hospital mortality (all *P* > .20).

## Discussion

This study suggests that RV strain parameters, particularly RV FWLS, are more strongly associated with the need for temporary MCS in patients with CS than conventional echocardiographic measures. These findings may have important implications for early risk stratification and clinical decision-making in critically ill cardiac patients.

### RV strain compared with conventional parameters

RV strain parameters showed stronger associations with temporary MCS use than commonly applied conventional measures (TAPSE and FAC). Severe RV free wall impairment demonstrated 10-fold increased odds of temporary MCS use. In contrast, TAPSE ≤10 mm and FAC ≤22% were not significantly associated with subsequent MCS. These observations are consistent with prior studies indicating that strain imaging provides more sensitive assessment of RV dysfunction than traditional parameters.^6,16,22–24^

Several factors may explain this finding. TAPSE reflects longitudinal motion of a single point at the tricuspid annulus, and does not capture the contribution of mid-ventricular, apical, and free wall segments. It is also influenced by passive motion from surrounding myocardial segments and loading conditions.^8,21,25,26^ Furthermore, TAPSE has known limitations in certain clinical contexts, including after cardiac surgery where compensatory circumferential shortening may preserve stroke volume despite reduced longitudinal motion.^7,21^ FAC, while incorporating both longitudinal and radial contraction, remains dependent on image quality and has limited reproducibility.^21^

In contrast, strain imaging quantifies active myocardial deformation and is less angle-dependent and less confounded by passive motion or geometric assumptions. RV FWLS has emerged as a sensitive measure of RV dysfunction across a wide spectrum of cardiovascular diseases.^9,27,28^ In specific patient categories, such as in acute myocarditis, the ability of strain parameters to detect subclinical dysfunction may explain their predictive value in identifying patients who will require temporary MCS.^29^

### Mechanisms of RV dysfunction

RV dysfunction is a recognized prognostic marker in CS across different aetiologies.^30^ Potential mechanisms include: (i) direct RV myocardial involvement from ischaemia, inflammation, or other primary processes; (ii) RV dysfunction secondary to LV failure through ventricular interdependence and increased RV afterload from pulmonary congestion; and (iii) alterations in septal mechanics affecting RV contractility.^7,31^ The strong performance of RV free wall strain, which specifically assesses the RV free wall independent of septal contribution, supports the importance of intrinsic RV myocardial dysfunction in determining the need for circulatory support.^9^

### Prognostic value and clinical outcomes

Severe RV FWLS impairment was associated with prolonged ICU and hospital length of stay, highlighting the clinical relevance of RV dysfunction for recovery trajectory and resource utilization. While prior studies have primarily focused on mortality and major cardiovascular events, our findings extend the literature by linking RV dysfunction to recovery-related outcomes such as length of stay.^6,15^

In contrast to some previous reports, RV functional parameters were not significantly associated with in-hospital mortality. This may reflect the heterogeneous and critically ill nature of our cohort, in whom mortality is strongly influenced by comorbidities, complications, and non-cardiac factors. Our population comprised of patients with CS of various aetiologies admitted to the ICU, whereas previous studies predominantly focused on more selected populations, such as patients with acute coronary syndrome, acute decompensated heart failure, or myocarditis. Notably, a proportion of patients had undergone resuscitation and sustained hypoxic brain injury, further contributing to mortality risk. Moreover, outcomes in CS may have improved in recent years with advances in MCS, particularly in high-volume centres with dedicated expertise. Notably, some patients were transferred from referring hospitals already receiving MCS, while others underwent immediate device implantation upon admission, with the first recorded TTE obtained after support initiation. According to our study design, these patients were excluded; however, they may represent a higher-risk subgroup with more advanced haemodynamic instability at presentation, which could have contributed to the absence of an observed mortality association. Finally, the study may have been underpowered to detect differences in mortality, given the relatively low number of events.

### Clinical implications

Prediction of temporary MCS requirements remains challenging despite existing risk scores.^2^

In this study, RV strain parameters showed stronger associations with temporary MCS use than severe LV systolic dysfunction, underscoring the importance of RV function in determining haemodynamic stability. This observation aligns with established understanding that RV failure after cardiac events is a serious complication associated with increased perioperative mortality, prolonged hospitalization, and worse outcomes.^2,32–34^

While RV FWLS showed a good discrimination with an AUC of 0.74, this parameter alone is not sensitive enough for prediction of temporary MCS, and a multiparametric approach incorporating clinical, haemodynamic, and imaging variables may be optimal for risk stratification. Consistent with this concept, a combined model incorporating RV FWLS, LVEF, and SOFA score demonstrated improved discriminative performance compared with individual parameters alone, achieving an AUC of 0.82. To further enhance clinical applicability, we developed an algorithmic risk stratification approach using CART analysis based on predefined dichotomized predictors. This approach identified RV FWLS as the primary discriminator of temporary MCS requirement, with subsequent refinement by SOFA score and LVEF. This approach yielded an interpretable risk stratification scheme, although external validation is required before clinical implementation.

RV FWLS provides incremental prognostic value beyond conventional RV parameters and LV function. Absolute RV FWLS values <20% are generally considered abnormal, with values <11% clearly indicating significant dysfunction.^8,9,27,28^ This may be particularly relevant for centres without on-site MCS capabilities, where early identification of patients likely to require advanced support could facilitate timely transfer and management to specialized centres.

From a practical perspective, RV strain assessment requires dedicated RV-focused imaging with clear visualization of the RV free wall myocardium, which can often be challenging at acute care setting. However, although measurement requires post-processing software, the technique has become increasingly standardized and reproducible, making it feasible in routine clinical practice.^9^

### Limitations and future directions

Several limitations warrant consideration. First, the cohort included patients with CS of diverse aetiologies; therefore, the findings may not be generalizable to each specific CS aetiology when considered independently. Moreover, the sample size was relatively small, which may have limited statistical power, particularly for less frequent outcomes.

In addition, the relatively high exclusion rate—primarily driven by the requirement for high-quality pre-MCS echocardiography suitable for RV strain analysis—may limit generalizability and introduce potential selection bias towards more stable patients able to undergo analysable imaging.

Importantly, the proposed predictive models and the CART-based algorithm were developed and tested within a single-centre cohort and lack external validation. Model performance may be overestimated in the derivation cohort and may not be directly transferable to other institutions or patient populations with different case mixes, management strategies, or imaging protocols. External validation in independent, multicentre cohorts is therefore essential before clinical implementation can be recommended.

Patients with severely reduced RV FWLS more frequently had heart failure–related CS, reflecting more advanced myocardial disease with biventricular involvement. This imbalance represents a limitation. However, RV FWLS remained independently associated with MCS requirement after adjustment for SOFA score and CS aetiology, suggesting that RV dysfunction reflects shock severity rather than aetiology alone.

Although RV strain measurements were available for all patients, TAPSE was missing in 13 cases, precluding a direct one-to-one comparison between strain-based and conventional RV functional parameters. In addition, vendor-specific variability in strain measurements, while improving, remains an important consideration, particularly for longitudinal assessment. Finally, the influence of loading conditions on strain measurements should be acknowledged, as RV strain parameters, although less load-dependent than conventional measures, are not entirely load-independent.

Future research should focus on validating RV strain cut-off values for predicting the need for temporary MCS across diverse patient populations. The development of multiparametric risk scores incorporating RV strain parameters, as well as the evaluation of strain-guided management strategies, may further clarify the clinical utility of RV strain assessment. Moreover, the potential role of serial RV strain measurements in monitoring response to therapy and guiding temporary MCS weaning warrants further investigation.

## Conclusion

This study suggests that impaired RV FWLS is associated with an increased likelihood of temporary MCS use and prolonged ICU and hospital length of stay in patients with CS. RV strain assessment may provide incremental information for early risk stratification and clinical decision-making in critically ill cardiac patients.

## Supplementary Material

xvag116_Supplementary_Data

## Declarations

### Data Availability

The data underlying this article are subject to data protection regulations (GDPR). Anonymized data are available from the corresponding author upon reasonable request.

### Pre-registered Clinical Trial Number

None supplied.
