# Supplementary material for: Right ventricular free-wall strain-based risk stratification for temporary mechanical circulatory support in cardiogenic shock
Source: ESC Heart Fail. 2026 Apr 27;13(3):xvag116. doi: 10.1093/eschf/xvag116 (PMC13188983; doi:10.1093/eschf/xvag116)
Supplement: xvag116_Supplementary_Data [file xvag116_supplementary_data.docx]

# **Supplementary material S1.: Baseline Characteristics in included versus excluded patients**

| **Parameter** | **Included patients (n=92)** | **Excluded patients (n=263)** | **p-value** | **Excluded patients with CS (n=240)** | **p-value** |
| --- | --- | --- | --- | --- | --- |
| **Baseline clinical status** |  |  |  |  |  |
| Age, years | 65 (52–76) | 64 (55–73) | 0.597 | 63 (55-73) | 0.435 |
| Male | 70 (76) | 202 (77) | 0.887 | 185 (77) | 0.885 |
| SOFA Score | 7 (4–11) | 10 (7–13) | <0.001 | 11 (7-13) | <0.001 |
| Lactate at admission, mg/dl | 24 (14–46) | 41 (21–81) | <0.001 | 45 (22-86) | <0.001 |
| **Mechanism of cardiogenic shock** |  |  |  |  |  |
| Cardiogenic shock | 92 (100) | 240 (91) | 0.001 | 240 (100) | N/A |
| LV failure | 79 (86) | 215 (82) | 0.622 | 215 (90) | 0.164 |
| RV failure | 41 (45) | 68 (26) | 0.002 | 68 (28) | 0.009 |
| Acute myocardial infarction cardiogenic shock | 22 (24) | 114 (43) | 0.001 | 114 (48) | <0.001 |
| Heart failure cardiogenic shock | 54 (59) | 67 (25) | <0.001 | 67 (28) | <0.001 |
| Secondary cardiogenic shock | 16 (19) | 42 (16) | 0.745 | 42 (18) | 1.000 |
| Post-cardiotomy cardiogenic shock | 0 (0) | 17 (7) | 0.009 | 17 (7) | 0.005 |
| **Classification of cardiogenic shock** |  |  |  |  |  |
| SCAI A | 2 (2) | 10 (4) | 0.738 | 10 (4) | 0.522 |
| SCAI B | 15 (16) | 16 (6) | 0.005 | 16 (7) | 0.011 |
| SCAI C | 43 (47) | 58 (22) | <0.001 | 58 (24) | <0.001 |
| SCAI D | 22 (24) | 74 (28) | 0.496 | 74 (31) | 0.227 |
| SCAI E | 10 (11) | 82 (31) | <0.001 | 82 (34) | <0.001 |
| **Outcomes** |  |  |  |  |  |
| MCS use | 31 (34) | 159* (61) | <0.001 | 156 (65) | <0.001 |
| In-hospital death | 31 (34) | 115 (44) | 0.110 | 107 (45) | 0.082 |
| 30-day mortality | 27/91 (30) | 107/248 (43) | 0.025 | 100/226 (44) | 0.022 |

*MCS without cardiogenic shock: 1 patient with type A aortic dissection, 1 with septic-haemorrhagic shock, 1 with septic shock and endocarditis.

Data are presented in median (Q1-Q3) and in n (%). For the comparison of categorical variables, Fisher’s exact test was applied, and for continuous variables, the Mann–Whitney U test was used. CS- Cardiogenic Shock, GCS – Glasgow Coma Sale, LV – left ventricle, MCS – mechanical circulatory support, RV – right ventricle, SCAI - Society for Cardiovascular Angiography and Interventions, SOFA - sequential organ failure assessment score.

# Supplementary material S2.: Baseline Characteristics in Patients with and without Severely reduced RV Longitudinal Free Wall Strain (RV FWLS) <11%

| **Parameters** | **RV FWLS ≥ 11%**  **(n=65)** | **RV FWLS < 11%**  **(n=27)** | **p-value** |
| --- | --- | --- | --- |
| **Baseline clinical status** |  |  |  |
| Age, years | 70 (59-79) | 55 (39-68) | <0.001 |
| Male | 45 (69) | 25 (93) | 0.017 |
| SOFA Score | 6 (3.5-11) | 8 (6-12) | 0.298 |
| GCS | 15 (11-15) | 15 (14-15) | 0.169 |
| Mechanical ventilation | 11 (17) | 3 (11) | 0.751 |
| Dialysis | 2 (3) | 2 (7) | 0.578 |
| Septic shock | 12 (18) | 2 (7) | 0.219 |
| Hemorrhagic shock | 1 (2) | 0 (0) | 1.000 |
| Pulmonary embolism | 4 (6) | 1 (4) | 1.000 |
| **Past medical history** |  |  |  |
| Arterial hypertension | 41 (63) | 7 (26) | 0.001 |
| Diabetes | 21 (32) | 6 (22) | 0.452 |
| Dyslipidemia | 35 (54) | 10 (37) | 0.173 |
| Coronary artery disease | 37 (57) | 8 (30) | 0.022 |
| Myocardial infarction | 27 (42) | 6 (22) | 0.097 |
| CABG | 5 (8) | 1 (4) | 0.667 |
| Other cardiac surgery | 7 (11) | 2 (7) | 1.000 |
| Pacemaker | 17 (26) | 8 (30) | 0.799 |
| Atrial fibrillation | 18 (28) | 5 (19) | 0.435 |
| COPD | 10 (15) | 4 (15) | 1.000 |
| **Drug therapy** |  |  |  |
| ARNI | 16 (25) | 11 (41) | 0.138 |
| ACE-I/ARB | 23 (35) | 3 (11) | 0.022 |
| Betablocker | 37 (57) | 15 (56) | 1.000 |
| Diuretics | 30 (46) | 11 (41) | 0.654 |
| SGLT2-I | 20 (31) | 13 (48) | 0.152 |
| MRA | 19 (29) | 12 (44) | 0.226 |
| **Laboratory** |  |  |  |
| Creatinine, mg/dl | 1.6 (1.2-2.3) | 1.8 (1.1-2.4) | 0.830 |
| Lactate at admission, mg/dl | 21 (14-51) | 30 (16-43) | 0.857 |
| Lactate during echocardiography, mg/dl | 21 (14-34) | 30 (12-47) | 0.487 |
| Troponine, ng/l | 98 (50-245)  (n=52) | 91 (40-485)  (n=17) | 0.884 |
| NTproBNP, ng/l | 9736 (3033-31592)  (n=50) | 13914 (5665-26541)  (n=19) | 0.510 |
| Bilirubin, mg/dl | 1.1 (0.6-1.5) | 1.0 (0.6-2.4) | 0.466 |
| **Mechanism of cardiogenic shock** |  |  |  |
| LV failure | 52 (80) | 27 (100) | 0.009 |
| RV failure | 24 (37) | 17 (63) | 0.037 |
| Biventricular failure | 16 (25) | 17 (63) | <0.001 |
| Acute myocardial infarction cardiogenic shock | 21 (32) | 1 (4) | 0.003 |
| Heart failure cardiogenic shock | 28 (43) | 26 (96) | <0.001 |
| Secondary cardiogenic shock | 16 (25) | 0 (0) | 0.002 |
| **Classification of cardiogenic shock** |  |  |  |
| SCAI A | 2 (3) | 0 (0) | 1.000 |
| SCAI B | 14 (22) | 1 (4) | 0.059 |
| SCAI C | 30 (46) | 13 (48) | 1.000 |
| SCAI D | 12 (18) | 10 (37) | 0.066 |
| SCAI E | 7 (11) | 3 (11) | 1.000 |

Data are presented in median (Q1-Q3) and in n (%). For the comparison of categorical variables, Fisher’s exact test was applied, and for continuous variables, the Mann–Whitney U test was used. ACE-I - Angiotensin-Converting Enzyme Inhibitor, ARB - Angiotensin II Receptor Blocker, ARNI - Angiotensinrezeptor-Neprilysin-Inhibitor, CABG – coronary artery bypass graft, COPD – chronic obstructive pulmonary disease, GCS – Glasgow Coma Sale, MRA - Mineralocorticoid Receptor Antagonist, NTproBNP - N-Terminal pro-B-type Natriuretic Peptide, LV – left ventricle, RV – right ventricle, SCAI - Society for Cardiovascular Angiography and Interventions, SGLT2-I - Sodium-Glucose Cotransporter 2 Inhibitor, SOFA - sequential organ failure assessment score.

# Supplementary material S3.: Clinical Outcomes in Patients with and without Severely reduced RV Longitudinal Free Wall Strain (RV FWLS) <11%

| **Parameters** | **RV FWLS ≥ 11%**  **(n=65)** | **RV FWLS < 11%**  **(n=27)** | **p-value** |
| --- | --- | --- | --- |
| **Mechanical circulatory support** |  |  |  |
| MCS use | 12 (18) | 19 (70) | <0.001 |
| Successful MCS weaning | 9/12 (75) | 8/19 (42) | 0.138 |
| Impella 5.5 | 6 (9) | 14 (52) | <0.001 |
| Successful Impella 5.5 weaning | 4/6 (67) | 7 /14 (50) | 0.642 |
| Impella CP | 7 (11) | 2 (7) | 1.000 |
| Successful Impella CP weaning | 5/7 (71) | 1/2 (50) | 1.000 |
| VA-ECMO | 6 (9) | 7 (26) | 0.050 |
| Successful VA-ECMO weaning | 5/6 (83) | 5/7 (71) | 1.000 |
| Temporary RVAD | 1 (2) | 3 (11) | 0.074 |
| Successful RVAD weaning | 0/1 (0) | 1/3 (33) | 1.000 |
| IABP | 0 (0) | 1 (4) | 0.293 |
| Successful IABP weaning | - | 1/1 (100) | - |
| Combined Impella and VA-ECMO*   - Impella CP - Impella 5.5 | 6 (9)  4 (6)  2 (3) | 5 (19)  0 (0)  5 (19) | 0.289 |
| **Intra-hospital outcomes** |  |  |  |
| Dialysis | 25 (38) | 11 (41) | 1.000 |
| Pacemaker implantation | 5 (8) | 3 (11) | 0.689 |
| Septic shock | 15 (23) | 5 (19) | 0.784 |
| Mechanical ventilation | 27 (42) | 14 (52) | 0.490 |
| Stroke | 1 (2) | 5 (19) | 0.008 |
| Intracranial bleeding | 2 (3) | 1 (4) | 1.000 |
| ICU stay, days | 8 (3.5-19) | 21 (11-35) | <0.001 |
| In-hospital stay, days | 14 (8-26) | 25 (14-41) | 0.003 |
| Discharge home from index hospital | 21 (32) | 10 (37) | 0.809 |
| Durable LVAD | 3 (5) | 9 (33) | <0.001 |
| Intra-hospital death | 24 (37) | 7 (26) | 0.344 |
| Death due to deterioration of cardiogenic shock | 18 (28) | 7 (26) | 1.000 |
| Death due to bleeding | 1 (2) | 0 (0) | 1.000 |
| Death due to sepsis | 5 (8) | 2 (7) | 1.000 |
| Death due to brain hypoxia | 4 (6) | 0 (0) | 0.317 |
| **Short-term outcome** |  |  |  |
| 30-day mortality | 20/64 (31) | 7 (26) | 0.802 |

*Patients receiving simultaneous VA-ECMO and Impella (ECMELLA) are presented as a separate subgroup. These patients are also included in the counts for individual devices (Impella CP, Impella 5.5, or VA-ECMO) for completeness.

Data are presented in median (Q1-Q3) and in n (%). For the comparison of categorical variables, Fisher’s exact test was applied, and for continuous variables, the Mann–Whitney U test was used. IABP – intra aortic balloon pump, LVAD – left ventricular assist device, MCS – mechanical circulatory support, RVAD – right ventricular assist device, VA-ECMO – veno-arterial extracorporeal membrane oxygenation
